# Preventive Treatment of Suppurative Inflammation of the Middle Ear

**Published:** 1888-05

**Authors:** R. O. Cotter

**Affiliations:** Macon, Ga.; Late Assistant to the Chair of Eye, Ear and Throat Diseases Atlanta Medical College


### PREVENTIVE TREATMENT OF SUPPURATIVE INFLAMMATION OF THE MIDDLE EAR.

BY R. O. COTTER, M. D., MACON, GA.
Late Assistant to the Chair of Eye, Ear and Throat Diseases Atlanta Medical College.

There is no disease in which the old adage, "an ounce of prevention is worth a pound of cure" is more *apropos* than this, and also, sad to say, in which the superior skill and management of the surgeon seems less appreciated. If promptly arrested the patient will usually imagine he had a simple earache, and which ought naturally to have been easily cured; and yet, after a by no means limited experience of over seven years in aural surgery, it is the writer's opinion that there is no disease of the ear which calls forth more careful judgment and discrimination, on part of the surgeon, than this one.

It is not the writer's purpose to give the extended symptoms of this, perhaps, most painful of all affections. We will assume the symptoms are pretty well known—that is, the subjective symptoms at any rate. We must be able to discriminate between the slighter earache, which usually tends to pass off within twelve to twenty-four hours, and usually comes on at night so frequently in children and young persons. Also the neuralgic earache, and of which the earache in children caused from carious teeth are another variety. To best illustrate, I will give the history of the management of one typical case of what might be "a sthenic case of acute inflammation of the middle ear with threatened suppuration and danger of perforated drum membrane." We read a great deal of *treatment* of these troubles *after* the drum membrane has ruptured and the trouble becomes both chronic and very troublesome. Now as to the *preventive* treatment. This patient, a healthy young man, who had tendency to throat trouble (pharyngitis) had a slight pharyngitis come on from exposure in the night air. The mouths of the eustachian tubes soon became irritable and painful, in the act of swallowing. In about four or five hours the pain gradually extended up into the right tympanum. In four hours more the pain was intense. The ear throbbing. The heart's pulsations thundering in the ear. Hearing intensely and painfully acute. He was seen at this point and a hypodermic of $\frac{1}{4}$ grain morphia injected in the arm, pain was somewhat duller in the course of three-fourths of an hour, but in two hours was as bad or worse than previous. Another hypodermic of morphia was given, and I may here state the morphia was kept up for a week four or five times daily. The patient's nervous system was so completely torn up by the agonizing pain I regarded the morphia as absolutely necessary.

By morning, say in about six or eight hours after the beginning of the pain in the ear, a douche of hot water, fully half gallon, was thrown into the ear, which was a great relief for a short while. Then a leech was applied to the tragus. The douche was used twice a day during the most of the painful period (a week). Leeches (one every day) were used. As to the drum membrane, by the second day it was very red and bulging, and in just about the proper condition to be punctured, a proceeding which, while it is so frequently advocated by writers cn the ear, is really very seldom resorted to by them. No doubt puncture at this juncture, in this case, *might* have liberated the pus or muco-pus and given relief. Yet I am averse to making a puncture in a drum membrane. My idea was to *prevent* suppuration and rupture, which would in all probability leave a troublesome and annoying chronic suppurative inflammation, and very probably a perforated drum membrane. The patient suffered a great deal for a week or ten days, but recovered with a sound drum membrane. At the end of ten days or two weeks his hearing in this ear, which was formerly five feet for the watch, was only *twelve inches*. and crackling, popping noises still persisted in the ear. These three troublesome symptoms were gradually relieved in the course of four or five weeks by inflation, by Politzer's method, employed about every other day. To sum up: My experience in these cases is that the leech is our very best remedy—use them freely. Next is the warm ear douche. Of course sufficient morphia must be used (and hypodermically to get its prompt action) to quiet the intense pain and nervous irritability. If full doses of morphia in the beginning do not seem to do great good, its very free later use seems to do harm by letting the patient down.

As soon as the patient is able to walk about, great care must be taken that he does not overtax his strength. Don't allow them to *tire* themselves, because nothing except, perhaps, taking cold, will be more apt to bring on a relapse.

In cold weather the patient should lie with a large piece of cotton batting bound over the affected ear. The idea is to keep the ear warm. It promotes the absorption of the lymph. Spirits of camphor warmed and dropped in the ear is soothing and beneficial; but nearly all the silly common household remedies, such as warm honey, sweet oil, etc., which soon become cold and lumpy, should be avoided.

